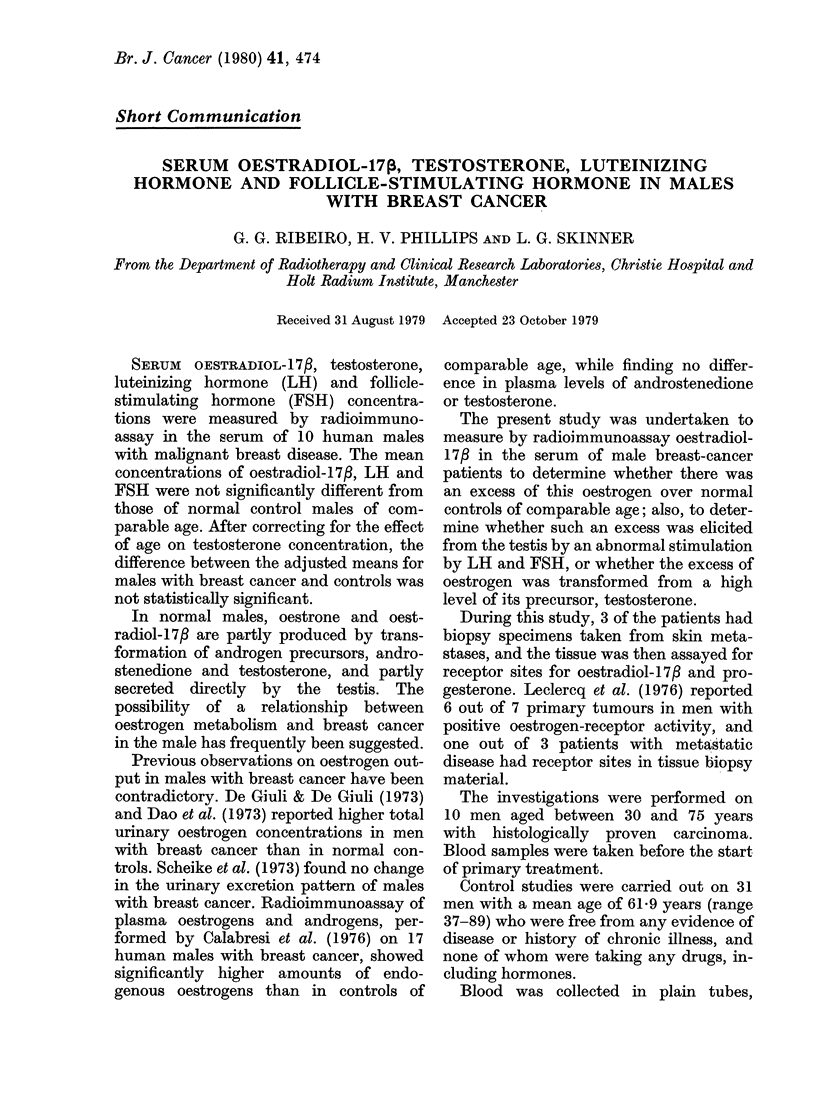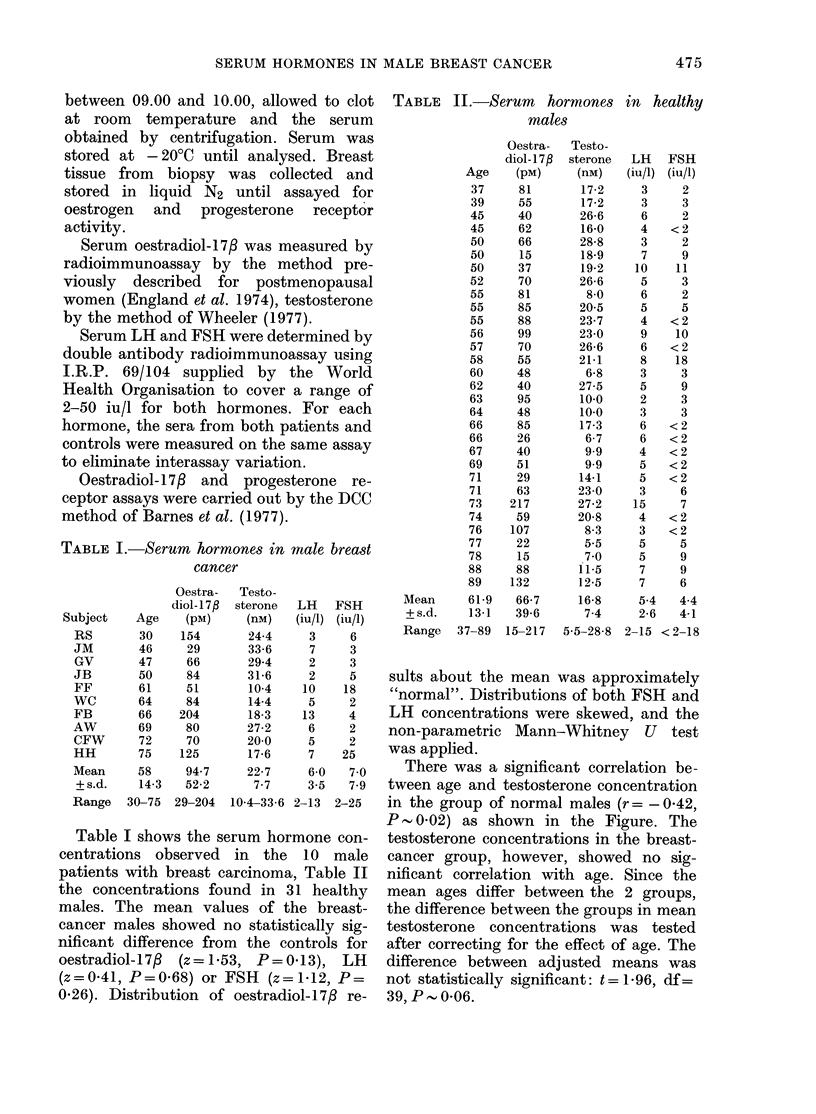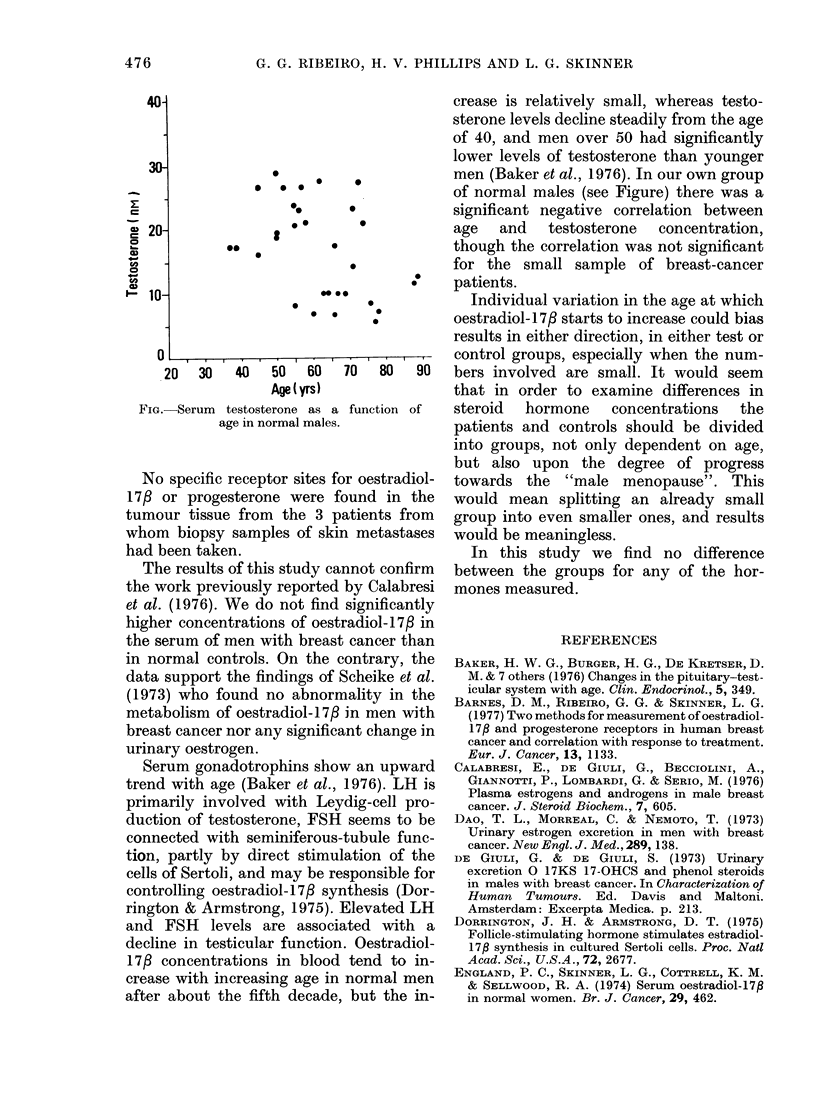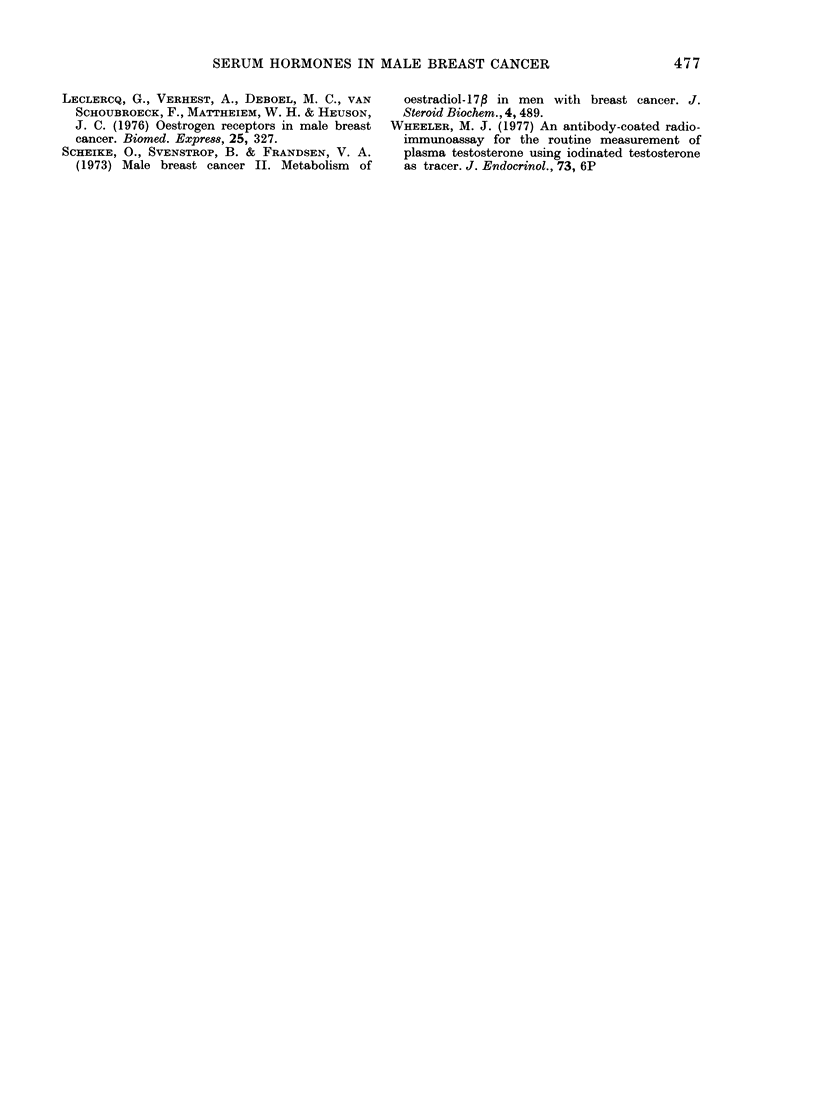# Serum oestradiol-17 beta, testosterone, luteinizing hormone and follicle-stimulating hormone in males with breast cancer.

**DOI:** 10.1038/bjc.1980.72

**Published:** 1980-03

**Authors:** G. G. Ribeiro, H. V. Phillips, L. G. Skinner


*Br. J. Cancer* (1980) **41**, 474

***Short Communication***

### SERUM OESTRADIOL-17β, TESTOSTERONE, LUTEINIZING HORMONE AND FOLLICLE-STIMULATING HORMONE IN MALES WITH BREAST CANCER

G. G. RIBEIRO, H. V. PHILLIPS AND L. G. SKINNER

*From the Department of Radiotherapy and Clinical Research Laboratories, Christie Hospital and Holt Radium Institute, Manchester*

Received 31 August 1979 Accepted 23 October 1979

SERUM OESTRADIOL-17β, testosterone, luteinizing hormone (LH) and follicle-stimulating hormone (FSH) concentrations were measured by radioimmunoassay in the serum of 10 human males with malignant breast disease. The mean concentrations of oestradiol-17β, LH and FSH were not significantly different from those of normal control males of comparable age. After correcting for the effect of age on testosterone concentration, the difference between the adjusted means for males with breast cancer and controls was not statistically significant.

In normal males, oestrone and oestradiol-17β are partly produced by transformation of androgen precursors, androstenedione and testosterone, and partly secreted directly by the testis. The possibility of a relationship between oestrogen metabolism and breast cancer in the male has frequently been suggested.

Previous observations on oestrogen output in males with breast cancer have been contradictory. De Giuli & De Giuli (1973) and Dao *et al.* (1973) reported higher total urinary oestrogen concentrations in men with breast cancer than in normal controls. Scheike *et al.* (1973) found no change in the urinary excretion pattern of males with breast cancer. Radioimmunoassay of plasma oestrogens and androgens, performed by Calabresi *et al.* (1976) on 17 human males with breast cancer, showed significantly higher amounts of endogenous oestrogens than in controls of comparable age, while finding no difference in plasma levels of androstenedione or testosterone.

The present study was undertaken to measure by radioimmunoassay oestradiol-17β in the serum of male breast-cancer patients to determine whether there was an excess of this oestrogen over normal controls of comparable age; also, to determine whether such an excess was elicited from the testis by an abnormal stimulation by LH and FSH, or whether the excess of oestrogen was transformed from a high level of its precursor, testosterone.

During this study, 3 of the patients had biopsy specimens taken from skin metastases, and the tissue was then assayed for receptor sites for oestradiol-17β and progesterone. Leclercq *et al.* (1976) reported 6 out of 7 primary tumours in men with positive oestrogen-receptor activity, and one out of 3 patients with metastatic disease had receptor sites in tissue biopsy material.

The investigations were performed on 10 men aged between 30 and 75 years with histologically proven carcinoma. Blood samples were taken before the start of primary treatment.

Control studies were carried out on 31 men with a mean age of 61·9 years (range 37–89) who were free from any evidence of disease or history of chronic illness, and none of whom were taking any drugs, including hormones.

Blood was collected in plain tubes, between 09.00 and 10.00, allowed to clot at room temperature and the serum obtained by centrifugation. Serum was stored at $-20°C$ until analysed. Breast tissue from biopsy was collected and stored in liquid $N_2$ until assayed for oestrogen and progesterone receptor activity.

Serum oestradiol-17$\beta$ was measured by radioimmunoassay by the method previously described for postmenopausal women (England *et al.* 1974), testosterone by the method of Wheeler (1977).

Serum LH and FSH were determined by double antibody radioimmunoassay using I.R.P. 69/104 supplied by the World Health Organisation to cover a range of 2–50 iu/l for both hormones. For each hormone, the sera from both patients and controls were measured on the same assay to eliminate interassay variation.

Oestradiol-17$\beta$ and progesterone receptor assays were carried out by the DCC method of Barnes *et al.* (1977).

TABLE I.—*Serum hormones in male breast cancer*

| Subject | Age | Oestradiol-17$\beta$ (pM) | Testosterone (nM) | LH (iu/l) | FSH (iu/l) |
|---|---|---|---|---|---|
| RS | 30 | 154 | 24·4 | 3 | 6 |
| JM | 46 | 29 | 33·6 | 7 | 3 |
| GV | 47 | 66 | 29·4 | 2 | 3 |
| JB | 50 | 84 | 31·6 | 2 | 5 |
| FF | 61 | 51 | 10·4 | 10 | 18 |
| WC | 64 | 84 | 14·4 | 5 | 2 |
| FB | 66 | 204 | 18·3 | 13 | 4 |
| AW | 69 | 80 | 27·2 | 6 | 2 |
| CFW | 72 | 70 | 20·0 | 5 | 2 |
| HH | 75 | 125 | 17·6 | 7 | 25 |
| Mean | 58 | 94·7 | 22·7 | 6·0 | 7·0 |
| ±s.d. | 14·3 | 52·2 | 7·7 | 3·5 | 7·9 |
| Range | 30–75 | 29–204 | 10·4–33·6 | 2–13 | 2–25 |

Table I shows the serum hormone concentrations observed in the 10 male patients with breast carcinoma, Table II the concentrations found in 31 healthy males. The mean values of the breast-cancer males showed no statistically significant difference from the controls for oestradiol-17$\beta$ ($z=1·53$, $P=0·13$), LH ($z=0·41$, $P=0·68$) or FSH ($z=1·12$, $P=0·26$). Distribution of oestradiol-17$\beta$ results about the mean was approximately "normal". Distributions of both FSH and LH concentrations were skewed, and the non-parametric Mann–Whitney $U$ test was applied.

There was a significant correlation between age and testosterone concentration in the group of normal males ($r=-0·42$, $P\sim0·02$) as shown in the Figure. The testosterone concentrations in the breast-cancer group, however, showed no significant correlation with age. Since the mean ages differ between the 2 groups, the difference between the groups in mean testosterone concentrations was tested after correcting for the effect of age. The difference between adjusted means was not statistically significant: $t=1·96$, df$=$39, $P\sim0·06$.

TABLE II.—*Serum hormones in healthy males*

| | Age | Oestradiol-17$\beta$ (pM) | Testosterone (nM) | LH (iu/l) | FSH (iu/l) |
|---|---|---|---|---|---|
| | 37 | 81 | 17·2 | 3 | 2 |
| | 39 | 55 | 17·2 | 3 | 3 |
| | 45 | 40 | 26·6 | 6 | 2 |
| | 45 | 62 | 16·0 | 4 | <2 |
| | 50 | 66 | 28·8 | 3 | 2 |
| | 50 | 15 | 18·9 | 7 | 9 |
| | 50 | 37 | 19·2 | 10 | 11 |
| | 52 | 70 | 26·6 | 5 | 3 |
| | 55 | 81 | 8·0 | 6 | 2 |
| | 55 | 85 | 20·5 | 5 | 5 |
| | 55 | 88 | 23·7 | 4 | <2 |
| | 56 | 99 | 23·0 | 9 | 10 |
| | 57 | 70 | 26·6 | 6 | <2 |
| | 58 | 55 | 21·1 | 8 | 18 |
| | 60 | 48 | 6·8 | 3 | 3 |
| | 62 | 40 | 27·5 | 5 | 9 |
| | 63 | 95 | 10·0 | 2 | 3 |
| | 64 | 48 | 10·0 | 3 | 3 |
| | 66 | 85 | 17·3 | 6 | <2 |
| | 66 | 26 | 6·7 | 6 | <2 |
| | 67 | 40 | 9·9 | 4 | <2 |
| | 69 | 51 | 9·9 | 5 | <2 |
| | 71 | 29 | 14·1 | 5 | <2 |
| | 71 | 63 | 23·0 | 3 | 6 |
| | 73 | 217 | 27·2 | 15 | 7 |
| | 74 | 59 | 20·8 | 4 | <2 |
| | 76 | 107 | 8·3 | 3 | <2 |
| | 77 | 22 | 5·5 | 5 | 5 |
| | 78 | 15 | 7·0 | 5 | 9 |
| | 88 | 88 | 11·5 | 7 | 9 |
| | 89 | 132 | 12·5 | 7 | 6 |
| Mean | 61·9 | 66·7 | 16·8 | 5·4 | 4·4 |
| ±s.d. | 13·1 | 39·6 | 7·4 | 2·6 | 4·1 |
| Range | 37–89 | 15–217 | 5·5–28·8 | 2–15 | <2–18 |

FIG.—Serum testosterone as a function of age in normal males.

No specific receptor sites for oestradiol-17$\beta$ or progesterone were found in the tumour tissue from the 3 patients from whom biopsy samples of skin metastases had been taken.

The results of this study cannot confirm the work previously reported by Calabresi *et al.* (1976). We do not find significantly higher concentrations of oestradiol-17$\beta$ in the serum of men with breast cancer than in normal controls. On the contrary, the data support the findings of Scheike *et al.* (1973) who found no abnormality in the metabolism of oestradiol-17$\beta$ in men with breast cancer nor any significant change in urinary oestrogen.

Serum gonadotrophins show an upward trend with age (Baker *et al.*, 1976). LH is primarily involved with Leydig-cell production of testosterone, FSH seems to be connected with seminiferous-tubule function, partly by direct stimulation of the cells of Sertoli, and may be responsible for controlling oestradiol-17$\beta$ synthesis (Dorrington & Armstrong, 1975). Elevated LH and FSH levels are associated with a decline in testicular function. Oestradiol-17$\beta$ concentrations in blood tend to increase with increasing age in normal men after about the fifth decade, but the increase is relatively small, whereas testosterone levels decline steadily from the age of 40, and men over 50 had significantly lower levels of testosterone than younger men (Baker *et al.*, 1976). In our own group of normal males (see Figure) there was a significant negative correlation between age and testosterone concentration, though the correlation was not significant for the small sample of breast-cancer patients.

Individual variation in the age at which oestradiol-17$\beta$ starts to increase could bias results in either direction, in either test or control groups, especially when the numbers involved are small. It would seem that in order to examine differences in steroid hormone concentrations the patients and controls should be divided into groups, not only dependent on age, but also upon the degree of progress towards the "male menopause". This would mean splitting an already small group into even smaller ones, and results would be meaningless.

In this study we find no difference between the groups for any of the hormones measured.

### REFERENCES

BAKER, H. W. G., BURGER, H. G., DE KRETSER, D. M. & 7 others (1976) Changes in the pituitary–testicular system with age. *Clin. Endocrinol.*, **5**, 349.

BARNES, D. M., RIBEIRO, G. G. & SKINNER, L. G. (1977) Two methods for measurement of oestradiol-17$\beta$ and progesterone receptors in human breast cancer and correlation with response to treatment. *Eur. J. Cancer*, **13**, 1133.

CALABRESI, E., DE GIULI, G., BECCIOLINI, A., GIANNOTTI, P., LOMBARDI, G. & SERIO, M. (1976) Plasma estrogens and androgens in male breast cancer. *J. Steroid Biochem.*, **7**, 605.

DAO, T. L., MORREAL, C. & NEMOTO, T. (1973) Urinary estrogen excretion in men with breast cancer. *New Engl. J. Med.*, **289**, 138.

DE GIULI, G. & DE GIULI, S. (1973) Urinary excretion O 17KS 17-OHCS and phenol steroids in males with breast cancer. In *Characterization of Human Tumours.* Ed. Davis and Maltoni. Amsterdam: Excerpta Medica. p. 213.

DORRINGTON, J. H. & ARMSTRONG, D. T. (1975) Follicle-stimulating hormone stimulates estradiol-17$\beta$ synthesis in cultured Sertoli cells. *Proc. Natl Acad. Sci., U.S.A.*, **72**, 2677.

ENGLAND, P. C., SKINNER, L. G., COTTRELL, K. M. & SELLWOOD, R. A. (1974) Serum oestradiol-17$\beta$ in normal women. *Br. J. Cancer*, **29**, 462.

LECLERCQ, G., VERHEST, A., DEBOEL, M. C., VAN SCHOUBROECK, F., MATTHEIEM, W. H. & HEUSON, J. C. (1976) Oestrogen receptors in male breast cancer. *Biomed. Express*, **25**, 327.

SCHEIKE, O., SVENSTROP, B. & FRANDSEN, V. A. (1973) Male breast cancer II. Metabolism of oestradiol-17β in men with breast cancer. *J. Steroid Biochem.*, **4**, 489.

WHEELER, M. J. (1977) An antibody-coated radioimmunoassay for the routine measurement of plasma testosterone using iodinated testosterone as tracer. *J. Endocrinol.*, **73**, 6P